# Diabetes‐Associated Major Limb Amputation in Solomon Islands: A National, 5‐Year Retrospective Study

**DOI:** 10.1002/wjs.70000

**Published:** 2025-07-14

**Authors:** Dylan M. Bush, Adrian Garcia Hernandez, Anita Pickard, Thomas H. Fitzpatrick, Rooney Jagilly, Micky Olangi, Michael Buin, Johanna Roth, Hendrick Kaniki, Jones Ghabu, Tony Quity, Alexandra L. Martiniuk

**Affiliations:** ^1^ Division of Biology and Medicine The Warren Alpert Medical School of Brown University Providence Rhode Island USA; ^2^ Data Science Institute, Fu Foundation School of Engineering and Applied Science Columbia University New York New York USA; ^3^ School of Public Health University of Sydney Sydney Australia; ^4^ World Health Organization Country Office for Solomon Islands Honiara Solomon Islands; ^5^ National Referral Hospital Solomon Islands Ministry of Health and Medical Services Honiara Solomon Islands; ^6^ Kilu'ufi Hospital Solomon Islands Ministry of Health and Medical Services Auki Solomon Islands; ^7^ Gizo Hospital Solomon Islands Ministry of Health and Medical Services Gizo Solomon Islands; ^8^ Department of City and Regional Planning University of California Berkeley California USA; ^9^ Helena Goldie Hospital Solomon Islands Ministry of Health and Medical Services Munda Solomon Islands; ^10^ Buala Hospital Solomon Islands Ministry of Health and Medical Services Buala Solomon Islands; ^11^ Dalla Lana School of Public Health The University of Toronto Toronto Canada; ^12^ The George Institute for Global Health Sydney Medical School The University of Sydney Barangaroo Australia

## Abstract

**Background:**

Solomon Islands is a Western Pacific nation with one of the highest diabetes prevalences in the world. The delivery of surgical care is challenging given the country's complex geography and limited healthcare resources. This retrospective study aims to quantify diabetes‐associated major limb amputation and to describe the characteristics of patients undergoing this procedure.

**Methods:**

Demographic, clinical, and surgical data were abstracted for patients who underwent major limb amputation secondary to diabetic infection. Summary statistics were gathered from this dataset. Additionally, patients' village names were abstracted, located from publicly available data, and mapped using ArcPro software. The geodesic distance between villages and the surgical centers performing amputation was calculated. Univariate and multivariate modeling was employed to assess the relationship between an a priori set of variables and several outcomes.

**Results:**

Over a 5‐year period (2018–2023), 401 amputations were performed on 356 patients with diabetic limb infections. Three hundred and five patients had medical records available for abstraction. The median age was 55 (range: 22–84 years), and 160 were male (52.5%). Trauma was the most common cause of ulceration (*N* = 135, 44.3%). Delayed presentation was common with 100 patients (32.8%) presenting more than 30 days after symptom onset. The mean Wagner score on presentation was 3.6 (STD: 0.8, range: 2–5). Most patients (*N* = 293, 96%) experienced surgical delays. Among all patients who underwent major limb amputation, 38 (10.7%) died prior to discharge. The mean linear distance between patient villages and surgical centers was 53.6 km. Greater distance was associated with more delayed presentation (Correlation Coeff: 0.14, *p* = 0.03) and higher Wagner scores (Kruskal–Wallis statistic: 10.7, *p* = 0.01). Multivariable logistic regression indicated that males were more likely to undergo above‐knee amputation as compared to females (OR: 2.13, *p* = 0.009). Further multivariable modeling demonstrated that male sex was correlated with higher wait times from presentation to amputation (*p* = 0.035).

**Conclusion:**

Major limb amputation is a costly and radical procedure which contributes to severe disability in the local context. Upstream interventions to manage diabetes and facilitate access to surgical care are likely to prevent limb loss.

## Graphical abstract

Solomon Islands is a Western Pacific nation with one of the highest diabetes prevalences in the world. The delivery of surgical care is challenging given the country's complex geography and limited healthcare resources. This retrospective study aims to quantify diabetes‐associated major limb amputation and to describe the characteristics of patients undergoing this procedure. Demographic, clinical, and surgical data were abstracted for patients who underwent major limb amputation secondary to diabetic infection. Summary statistics were gathered from this dataset.

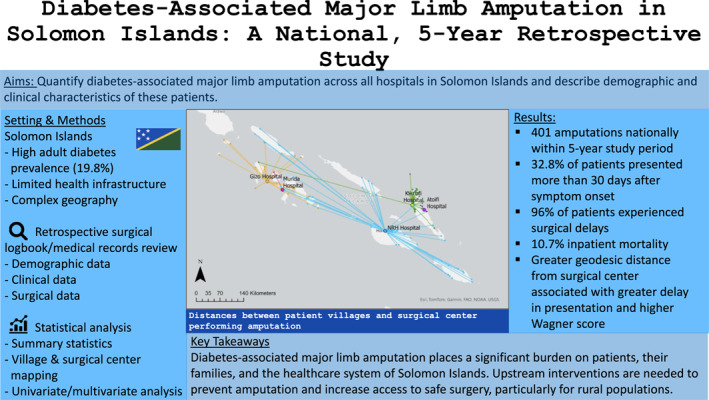

## 1 Introduction

Diabetes is a significant health issue in Solomon Islands, a country with the 13th highest diabetes prevalence globally at 19.8% for individuals aged 20–79 years [1]. Diabetes is the third leading cause of death [2] in Solomon Islands and there is a high prevalence of diabetes‐associated complications and co‐morbidities in Pacific Island nations [3].

Major limb amputation (MLA) secondary to diabetic infection is a costly procedure that necessitates extensive pre‐operative, post‐operative, and rehabilitative management, placing a significant burden on healthcare systems [4]. Internationally, the pooled 5‐year mortality rate for diabetes‐related MLA is reported between 56.6%–80% [4, 5] with secondary amputations common, contributing further to morbidity and mortality [6]. Win Tin et al. surveyed 160 Solomon Islanders with diabetes and found a high prevalence of microalbuminuria (36%), diabetic retinopathy (40%), and lower extremity neuropathy (23%) [3].

Despite a high incidence of diabetes‐associated lower limb amputations in limited resource countries [7, 8], including in Pacific Island nations [9, 10, 11, 12], the overall incidence of diabetes‐associated MLA has never been reported in Solomon Islands.

Solomon Islands has a tiered healthcare system consisting of nurse aid posts, rural health centers, area health centers, general hospitals, and the National Referral Hospital (NRH)—the nation's only tertiary care center [13, 14, 15]. This system faces challenges in delivering preventive and continuing diabetes care due to limitations in health workforce, and frequent shortages of surgical supplies and medications—including insulin, which contributes to poor glycemic control and diabetes complications [3, 16, 17]. Socioeconomic disadvantage, physical inactivity and poor diet quality [18, 19], distance from diabetic foot care services [20, 21, 22], poorly controlled blood glucose, smoking, heavy alcohol consumption, and a BMI over 32 kg/m^2^ increase diabetes‐associated amputation risk, and are all prevalent health concerns in Solomon Islands [23, 24, 25, 26].

Cases of diabetes‐related foot disease in Pacific Island nations are primarily managed by general or orthopedic surgeons, or general medicine physicians due to a lack of subspecialty services, including vascular surgery [16]. Vascular surgery interventions have been shown to be crucial for limb salvage in other LMICs as part of the diabetes care continuum [27, 28]. Diabetic foot ulcer patients in LMICs often present at advanced stages, as seen in Figure S1, due to delays in diagnosis, limited preventative care and insufficient wound care services [29, 30, 31].

In Solomon Islands, amputations are performed by general surgeons. Surgical services are primarily concentrated at the National Referral Hospital. A small number of provincial hospitals and area health centers also provide limited surgical services, with only a few provincial hospitals capable of performing MLA. In Solomon Islands, this procedure is typically performed under spinal anesthesia either with or without a tourniquet depending on surgeon preference. Surgeons use sharp dissection to transect nerves and soft tissue along with electrocautery or suture ligature for hemostasis depending on resource availability. Bones are transected via manual bone saw. Amputations are typically closed through primary closure or, more commonly, via delayed primary closure to allow for infection control. Currently, negative wound pressure therapy and advanced closure techniques such as extracellular matrix scaffolds are unavailable in Solomon Islands.

Global data on diabetes‐associated amputations are inconsistent [1], with significant gaps regarding data on diabetes‐associated amputation in LMICs [32, 33, 34, 35]. Reliable data on diabetic amputations can better inform resource allocation, preventive strategies and interventions, and follow‐up care of people with diabetic amputations. This study aimed to measure the incidence of diabetes‐associated MLAs in Solomon Islands and to describe the clinical characteristics and surgical outcomes of patients undergoing these procedures.

## 2 Methods

This study was approved by the Solomon Islands Health Research Ethics Review Board (HRE004/21).

### 2.1 Inclusion and Exclusion Criteria

Patients were deemed eligible for inclusion if they underwent MLA due to a diabetes‐associated infection in Solomon Islands between January 2018 and January 2023. MLA was defined as amputations of the lower extremity proximal to the ankle and amputations of the upper extremity proximal to the wrist.

Patients were excluded if they underwent amputation due to a non‐diabetic etiology, underwent minor amputation alone, or did not have medical records available. The researchers decided to exclude patients who underwent minor amputation secondary to diabetic infection as these procedures are sometimes performed on an outpatient basis and thus patients may lack in‐hospital medical records.

### 2.2 Data Collection

Prospectively maintained operating theater logbooks were retrospectively reviewed. Researchers gathered data from all 6 surgical centers that performed MLA within the study period.

Data from surgical logbooks including age, sex, diagnosis, date of surgery, procedure type, and side of amputation were entered into an electronic database. The medical records of patients who underwent MLA were obtained from the respective records department of each surgical center. If records were not locatable by patient name, date of birth, and medical record number, a member of the research team manually searched through all medical records entered during the year when the amputation took place. If records were not locatable following this secondary search, then data were not entered, and the patient was excluded from analysis.

### 2.3 Variables

Patient charts were reviewed for demographics, admission information, clinical characteristics, surgical management, delays, and postoperative outcomes. Delays were abstracted from provider narratives written in the medical chart and encompassed both patient and provider‐reported delays in presentation to medical care, and to definitive surgical management. Outcomes included date of discharge and date of death, if applicable.

For patients with multiple MLAs during the study period, all amputation dates and types were entered into the database; however, perioperative and surgical data were only entered for the most proximal amputation. In the case that a patient had bilateral amputations of the same nature and data were available for both admissions, the most recent amputation was entered.

A full list of variables and variable categories is presented in Table S1.

### 2.4 Geographic Information System Analysis

Village locations, as recorded in patients' medical charts, were obtained from publicly available data and were mapped using ArcPro software. ArcPro's Near (Analysis) calculated the geodesic distance between patients' villages and the hospital where amputation occurred.

### 2.5 Statistical Analysis

Statistical analysis was performed using Python 3.9.6 and R 4.3.1. Descriptive analysis was used to count the frequency (%) of categorical variables and the mean or median of continuous variables. Range, standard deviation (SD), and interquartile range (IQR) were reported where appropriate.

Outcome measures for univariate and multivariate regression analysis included wait time for amputation, Wagner score on presentation, and amputation type.

Univariate analysis was used to test for associations between distance from surgical centers and wait time for amputation, Wagner score, and amputation type (above‐knee as compared to below‐knee). Additionally, univariate analysis was used to assess the association between an a priori list of both clinical and demographic variables and the type of amputation performed.

Multivariable regression modeling, controlling for referral status and province of origin, was used to assess the relationship between sex and amputation type. Another multivariable regression model that controlled for referral status and province of origin assessed the association between sex and wait times from presentation to amputation.

## 3 Results

### 3.1 Diabetic Major Limb Amputation

**FIGURE 1:**
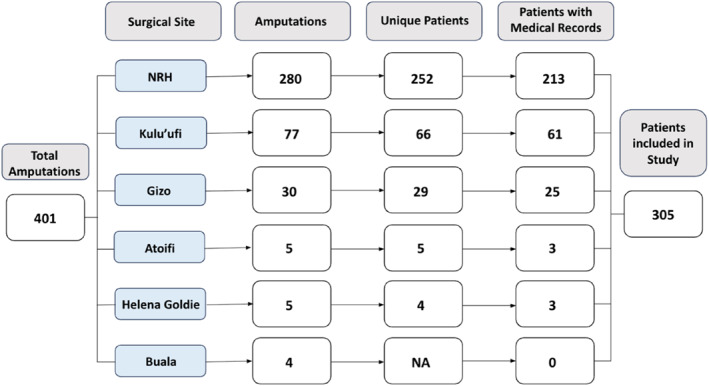
Flow chart of patient inclusion across surgical centers.

During the study period, 401 MLAs were performed on patients with diabetes‐associated limb infections at 6 hospitals. A flow chart illustrating the number of amputations and the number of patients included in this study is displayed in Figure 1. Most amputations were performed at the NRH (*N* = 280, 69.8%), with smaller numbers performed at provincial hospitals including Kilu'ufi Hospital (*N* = 77, 19.2%), Gizo Hospital (*N* = 30, 7.5%), Atoifi Adventist Hospital (*N* = 5, 1.2%), Henela Goldie Hospital (*N* = 5, 1.2%), and Buala Hospital (*N* = 4, 1.0%).

Amputations were performed on 356 unique patients. Three hundred and five (85.7%) had medical records available for abstraction at five surgical centers. Buala Hospital did not have medical records available for abstraction. Amputations, organized by surgical center and type, for patients with medical records is reported in Table 1. The most common procedure was below‐the‐knee amputation (BKA) (*N* = 223, 73.1%), followed by above‐knee (AKA) (*N* = 75, 24.6%), above‐elbow (*N* = 5, 1.6%), forearm (*N* = 1, 0.33%) and through‐knee (*N* = 1, 0.33%). One hundred and sixty‐two amputations were right‐sided (53.1%). The median age for this cohort was 55 (range: 22–84 years), and 160 were male (52.5%). Additional demographics are reported in Table 2. Among patients with data entered for smoking history (*N* = 239), 89 (37.3%) reported a history of tobacco use. One hundred and fifty‐eight patients (51.8%) undergoing MLA had one or more previous amputation, either major or minor, in the study period.

**TABLE 1:** Number of amputations by surgical center and type.^a^

Surgical center	Count	Percentage (*N* = 305) (%)
Atoifi Hospital	3	0.98
Above‐elbow	1	0.33
Below‐knee	2	0.66
Helena Goldie Hospital	3	0.98
Below‐knee	3	0.98
Gizo Hospital	25	8.20
Above‐knee	5	1.64
Below‐knee	20	6.56
Kulu'ufi Hospital	61	20.00
Above‐elbow	3	0.98
Above‐knee	9	2.95
Below‐knee	49	16.07
National Referral Hospital	213	69.84
Above‐elbow	1	0.33
Above‐knee	61	20.00
Below‐knee	149	48.85
Forearm	1	0.33
Through‐knee	1	0.33
Total	305	100.00

^a^ Although 401 amputations were performed during the study period, Table 1 only includes amputations with available medical records.

**TABLE 2:** Summary of patient demographics.

Variable	Count	Percentage (*N* = 305) (%)
Sex		
Female	145	47.54
Male	160	52.46
Smoking status		
Current	74	24.26
Former	15	4.92
Never	150	49.18
Unknown	66	21.64
Prior amputation during study period		
Yes	158	51.80
No	147	48.20
Total	305	100.00

In terms of admission, most patients (*N* = 185, 60.7%) were admitted to the Emergency Department. Only 38 were admitted directly to the surgical ward (12.5%). Admissions data summarized by department are displayed in Table S2. One hundred and seventy‐eight patients (58.4%) had a record of referral from another medical center in their medical chart. Most referrals came from provincial hospitals (*N* = 65, 36.5%), other departments within the NRH (*N* = 35, 19.7%), area health centers (*N* = 25, 14.0%), and rural health centers (*N* = 25, 14.0%). Among patients who were referred from another medical center, the emergency department was again the most common admitting department (*N* = 112, 62.9%) with only 17 patients (9.6%) being admitted directly to the surgical ward. Referrals were sent from 55 clinics, hospitals, and departments. The highest volume of referrals by referring site came from the Diabetes/NCD Clinic at the NRH (*N* = 34, 19.1%), Kirakira Hospital (*N* = 16, 9.0%), and Good Samaritan Hospital (*N* = 13, 7.3%).

Among all patients with medical records, 303 (99.3%) had data concerning the date of their initial diabetes diagnosis. Overall, 286 patients (93.8%) were diagnosed prior to their present admission while 17 patients (5.6%) were unaware that they had diabetes before presenting with a diabetic limb infection. Among patients with known diabetes, 230 (80.4%) were asked about diabetes medication adherence. A minority of these patients (*N* = 50, 21.7%) reported compliance with medication. The median duration of diabetes mellitus diagnosis to present admission was 6.1 years (STD: 5.7 years). Regarding diabetes medications, 141 patients (49.3%) were prescribed metformin, 48 (16.8%) were prescribed glibenclamide, and 11 (3.9%) were prescribed glipizide. The median blood glucose on admission was 20.0 (STD: 7.13). Additionally, 69 patients (22.6%) had an existing diagnosis of hypertension.

A Wagner score based on clinical presentation at time of admission was calculated for 221 patients (72.5%). The mean Wagner score was 3.6 (STD: 0.8, range: 2–5), consistent with deep tissue infection with possible osteomyelitis or tendonitis. The most common presenting symptoms included fever (*N* = 201, 65.9%), pain (*N* = 124, 40.7%), and loss of appetite (*N* = 124, 40.7%). The initial cause of ulceration was known and recorded for 160 patients (52.5%). Trauma was the most reported cause of ulceration (*N* = 135, 44.3%). A full list of initial causes of ulceration is reported in Table S3. Most patients (*N* = 242, 79.3%) reported waiting longer than 7 days from the start of symptoms before presenting to medical care, with 100 patients (32.8%) waiting longer than 30 days. After arriving at a medical facility, nearly all patients (*N* = 293, 96%) experienced surgical delays. Categories of surgical delay are reported in Table 3. The most common cause of surgical delay was unavailability of the operating theater or of surgical staff (*N* = 180, 59.0%). Over the course of their hospital stay, most patients (*N* = 300, 98.4%) demonstrated under‐treated diabetes, defined as having two blood sugar level (BSL) readings above 8.5 mmol/L, excluding BSL measured during the 48‐h stabilization period following admission.

**TABLE 3:** Surgical delays listed by type.

Surgical delay type	Count	Percentage (*N* = 305) (%)
Theater delay	180	59.02
Poor laboratory results	172	56.39
Transfusion not available	128	41.97
Provincial referral	96	31.48
Patient decision	80	26.23
Intra‐provincial referral	52	17.05
Patient relative decision	33	10.82
Kastom^a^	25	8.20
Accidental eating	24	7.87
Cardiac issues	16	5.25
Too much swelling	2	0.66
High blood pressure	2	0.66
Positive for COVID‐19	2	0.66
Unable to anesthetize	2	0.66
Patient too weak	1	0.33
Stroke	1	0.33
Low blood pressure	1	0.33

^a^ Kastom refers to patient preference for traditional medicine and delays due to refusal of surgical intervention.

Microbiological results for this cohort have been reported previously [36]. Pre‐operatively, 304 patients (99.7%) were treated with antibiotics. The most prescribed antibiotics included Cloxacillin (*N* = 293, 96.1%), Metronidazole (*N* = 282, 92.5%), and Ceftriaxone (*N* = 73, 23.9%). Prior to MLA, 206 patients (67.5%) underwent a surgical intervention, including debridement (*N* = 162, 53.1%), fasciotomy (*N* = 13, 4.3%), or minor amputation (*N* = 124, 40.7%). Additionally, 50 patients (16.4%) underwent a more distal major amputation, including below‐the‐knee amputation, prior to the terminal MLA. Pre‐operative blood transfusion was reported for 207 patients (67.9%). The mean volume of pre‐operative blood transfusion was 2.63 L (SD:1.73). Pre‐operative bloodwork showed several abnormalities including hyponatremia, anemia, hyperphosphatemia, and neutrophilic leukocytosis. Pre‐operative bloodwork results are reported in full in Table S4.

Because of challenges in administering general anesthesia in Solomon Islands, nearly all patients (*N* = 297, 97.4%) were placed under spinal anesthesia for their amputation. Although most patients were awake for the entirety of their surgery, some were administered ketamine; however, records regarding ketamine administration during surgery were incomplete.

Forty‐nine patients (16.1%) experienced postoperative complications. The most frequently reported post‐operative complications included hypotension (*N* = 36, 11.8%), excessive bleeding (*N* = 10, 3.3%), altered consciousness (*N* = 8, 2.6%), and shock (*N* = 4, 1.3%). All four of these patients who experienced shock died during their hospital stay.

Death records existed both for patients with and without medical records. Among all patients (*N* = 356), 38 (10.7%) died prior to discharge. Sepsis was the most common cause of death (*N* = 20, 52.6%). A cause of death was not recorded for 9 patients. Six patients died within 48 h of amputation. Among the six patients who died within 48 h, sepsis was a contributing factor in three cases. The median duration of admission at a surgical center was 43 days.

### 3.2 Geographical Access to Surgical Centers

Geographical village coordinates were available for 280 patients. The mean linear distance from a surgical center was 53.6 km. Of these 280 patients, 265 (94.6%) had both location and clinical data available for analysis. These patients were used for location‐associated statistical analysis as detailed below.

**FIGURE 2:**
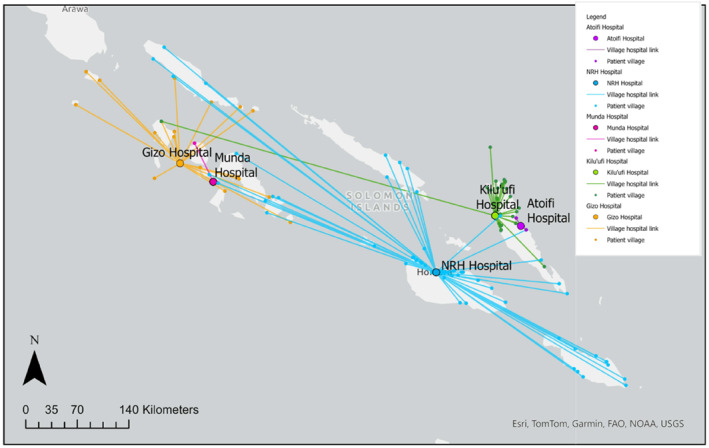
Distances between patient villages and surgical centers that performed major limb amputation.

Data regarding geodesic distance from surgical centers is summarized by province in Table 4. Average distances varied widely between surgical centers with patients presenting to Gizo Hospital traveling the farthest (Avg: 84.6 km) and patients presenting to Atoifi Hospital traveling the shortest distance on average (Avg: 8.1 km). Distances also varied widely among the patients treated at each surgical center. The largest spread in distance was observed at the NRH, where the smallest distance measured was 0.3 km and the largest was 477.2 km. Distances between patients' villages and the surgical center where they underwent limb amputation are mapped in Figure 2.

**TABLE 4:** Geodesic distance between patient villages and surgical centers performing amputation in kilometers.

Surgical center	Mean distance	Minimum distance	Maximum distance
Atoifi Hospital	8.1	3.15	12.4
Gizo Hospital	84.6	2.5	177.0
Kilu'ufi Hospital	36.4	2.0	466.6
Helena Goldie Hospital	21.8	3.6	58.0
National Referral Hospital	55.5	0.3	477.2

### 3.3 Significant Risk Factors

Greater distance from a surgical center was significantly associated with longer wait times between the onset of symptoms and presenting to a healthcare facility (Correlation Coeff: 0.14, *p* = 0.03). Additionally, median distance from a surgical center was associated with a higher Wagner score, corresponding to more advanced gross pathology on presentation (Kruskal Wallis statistic: 10.7, *p* = 0.01). Interestingly, distance did not influence whether patients underwent BKA as compared to AKA (Kruskal‐Wallis statistic: 0.6, *p* = 0.4).

Univariate analysis was conducted to determine the possible relationship between independent variables and the likelihood of undergoing AKA versus BKA as the terminal procedure. These tests shows that higher white blood cell counts (*p* = 0.0002) and being male (*p* = 0.016) were correlated with having an AKA as the terminal procedure.

A multivariable logistic regression was conducted to determine the relationship between sex and terminal procedure when controlling for referral status and province of origin. This test showed that males were more than twice as likely to undergo AKA than females (odds ratio = 2.13, *p* = 0.009). Likewise, a multivariable linear regression controlling for referral status and province of origin showed that male sex was correlated with higher wait times from presentation to terminal procedure (*p* = 0.035).

## 4 Discussion

This is the first national study of diabetic amputation in Solomon Islands. The sample was demographically diverse, including patients from all nine provinces who underwent MLA at all six hospitals performing such procedures during the study period. Results align with high incidence of lower limb amputations in other Pacific Island nations [9, 10, 11, 12], and increasing rates of diabetes‐associated MLA in resource limited countries [7, 8].

Findings highlight significant gaps along the diabetes care continuum in Solomon Islands, including poor self‐management, late presentation, delayed referral along the levels of care, sub‐optimal inpatient medical management, delayed surgical intervention, and limited access to follow‐up care. Inadequate management, and insufficient patient education for self‐care are associated with late presentation for diabetic complications and poor patient outcomes [8, 9, 12, 29, 37]. As reported above, only 21.7% of patients who were asked about compliance reported compliance with diabetes medications. Even within the hospital setting, we found that 98.4% of patients had under‐controlled diabetes. Prior research from the Pacific region, including Solomon Islands, has suggested that preference for traditional treatments and insufficient knowledge about diabetes and diabetic foot care are important patient‐level factors leading to amputation [38]. These health system and patient‐level challenges in Solomon Islands, including poor self‐management of diabetes, parallels trends in other Pacific Island countries and territories [39], and LMICS more broadly [3, 16, 17].

Our data show many patients presented late and faced further delays in receiving essential surgical interventions. Whilst 60.7% of patients were admitted through the Emergency Department, it is noted this is the primary entry pathway into the hospital. Despite severe wound classification scores and systemic infection symptoms, only 10.5% (*N* = 32) were admitted directly to the surgical ward, highlighting the need for fast‐track referral pathways for timely limb‐salvaging surgery. Similarly, innovative and culturally appropriate techniques to expand the reach of diabetes‐related health promotion and follow‐up care for diabetic amputees may improve health‐seeking behavior, lessen readmission, and reduce repeated amputation.

Extensive work was required to collect and manage the data for this study. Significant medical record gaps remain, as highlighted by the data reported here. Whilst the findings provide comprehensive insight into the current diabetic amputation and surgery systems in Solomon Islands, there is a need to strengthen facility and national data management systems.

### 4.1 Limitations

This retrospective study only captures data on patients who presented to a surgical center, introducing a survival bias. Noting the lengthy delays in presentation described here, a proportion of patients with diabetic limb infections may have died prior to reaching a surgical center.

Currently, all hospitals in Solomon Islands rely on paper medical charts, and a new chart under a different medical record number is generated for each re‐admission. The absence of a functional, longitudinal health records system precluded assessment of long‐term outcomes such as post‐amputation mortality, recovery, and rehabilitation. Limited laboratory capacity and the absence of hemoglobin A1C testing in Solomon Islands also impacted researchers' ability to assess long‐term glucose control among diabetic patients presenting for MLA.

Calculation of actual distance traveled by patients to reach surgical centers is not possible in Solomon Islands given the infinite number of routes over footpaths, unmapped roads, and waterways. Geodesic distances likely represents and under‐estimation of true distance travelled and may have biased results toward patients living nearer to a surgical center.

## 5 Conclusion

This study highlights the challenges faced by surgical systems in a resource‐limited country to address end‐stage complications of diabetes and diabetic foot. However, the burden incurred by diabetic complications on surgical services in Solomon Islands is also a consequence of upstream systems failures. A stronger continuum of care throughout the entire pre‐ and postoperative pathway, including primary, secondary, tertiary and rehabilitative care, would improve patient outcomes, and understanding of patient outcomes post‐discharge. Further investment in diagnosis and diabetes management at the community level may reduce the rate of end‐stage complications leading to infection, amputation and death. Additionally, future research on upstream interventions, including limb‐sparing surgical interventions, is needed.

## Author Contributions

**Dylan M. Bush:** conceptualization, project administration, investigation, methodology, data curation, formal analysis, writing – original draft, writing – review and editing. **Adrian Garcia Hernandez:** data curation, formal analysis, writing – original draft. **Anita Pickard:** writing – original draft, writing – review and editing, visualization. **Thomas H. Fitzpatrick:** writing – original draft, writing – review and editing. **Rooney Jagilly:** methodology, supervision, writing – review and editing. **Micky Olangi:** resources, writing – review and editing. **Michael Buin:** resources, writing – review and editing. **Johanna Roth:** writing – original draft, writing – review and editing, visualization. **Hendrick Kaniki:** resources, writing – review and editing. **Jones Ghabu:** methodology, writing – review and editing. **Tony Quity:** resources, writing – review and editing. **Alexandra L. Martiniuk:** methodology, supervision, validation, writing – review and editing.

## Conflicts of Interest

The authors declare no conflicts of interest.

## Supporting information

Supporting Information S1

## Data Availability

The data that support the findings of this study are available from the corresponding author upon reasonable request.
