# Supplementary material for: Diabetes‐Associated Major Limb Amputation in Solomon Islands: A National, 5‐Year Retrospective Study
Source: World J Surg. 2025 Jul 14;49(8):2266–74. doi: 10.1002/wjs.70000 (PMC12338441; doi:10.1002/wjs.70000)
Supplement: Supplementary file 1 — Supporting Information S1 [file WJS-49-2266-s001.docx]

**Supplement**

**Supplemental Figure 1**. Extensive diabetic wound of a Solomon Islands patient shortly after presentation and prior to surgical intervention.


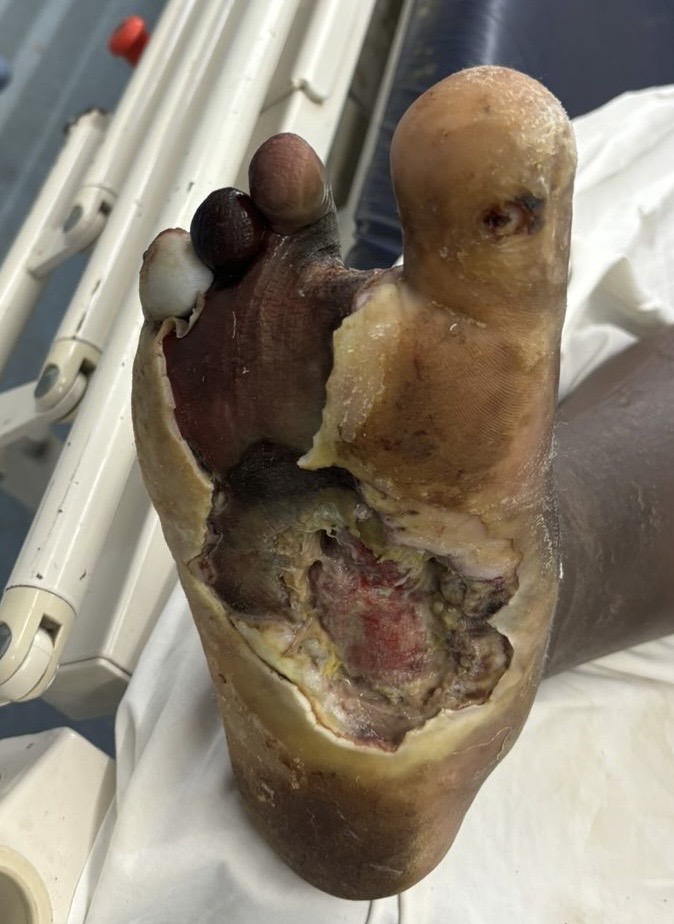


**Supplemental Table 1.** Variables collected from patient medical records.

| **Domain** | **Variable Name** | **Description** | **Type** |
| --- | --- | --- | --- |
| Demographics | Sex | Patient's biological sex | Categorical |
| Demographics | Age | Age at time of admission | Continuous |
| Demographics | Religion | Patient-reported religious affiliation | Categorical |
| Demographics | Occupation | Patient-reported primary occupation | Categorical |
| Demographics | Village of origin | Patient's village of residence | Categorical |
| Demographics | Province of origin | Province corresponding to patient's home village | Categorical |
| Admission Details | Date of admission | Calendar date of initial hospital admission | Date |
| Admission Details | Admitting department | Hospital ward to which the patient was admitted | Categorical |
| Admission Details | History of referral | Documentation of referral from outside facility | Binary |
| Clinical Features | Comorbidities | Documented chronic medical conditions | Categorical |
| Clinical Features | Prior amputation | Previous limb amputation noted in medical chart | Binary |
| Clinical Features | Symptoms at presentation | Chief complaints as documented at admission | Categorical |
| Clinical Features | Wagner score | Ulcer severity score | Ordinal |
| Clinical Features | Vital signs | Temperature, pulse, blood pressure, respiratory rate | Continuous |
| Clinical Features | Mechanism of injury | Etiology of foot injury | Categorical |
| Clinical Features | Time to presentation | Duration from symptom onset to presentation | Continuous |
| Clinical Features | Current medications | Documented medications prior to admission | Categorical |
| Clinical Features | Medication adherence | Patient compliance with prescribed medications | Binary |
| Clinical Features | Pre- and post-operative bloodwork | Lab values including CBC, glucose, etc. | Continuous |
| Surgical Management | Procedure type | Amputation level (e.g. above-knee, below-knee, above-elbow, below-elbow) | Categorical |
| Surgical Management | Procedure location | Anatomical side of amputation (e.g. left, right) | Categorical |
| Surgical Management | Anaesthetic type | Anaesthetic used (general, spinal, local) | Categorical |
| Surgical Management | Blood transfusion | Amount (units) and timing relative to surgery | Continuous / Date |
| Surgical Management | Intra-operative complications | Documented complications during procedure | Binary / Text |
| Surgical Management | Post-operative complications | Immediate complications following surgery | Categorical |
| Delays | Delay in presentation | Provider-reported or patient-reported delays in seeking care | Binary / Narrative |
| Delays | Delay in surgical management | Delay from admission to definitive amputation | Continuous |
| Outcomes | Date of discharge | Date of patient's discharge from hospital | Date |
| Outcomes | In-hospital mortality | Date of death if patient died during admission | Binary / Date |

*Note: Certain variable categories (e.g., pre- and post-operative bloodwork) include multiple individual data points.

**Supplemental Table 2.** Admissions by admitting department.

| **Admitting Department** | **Count** | **Percentage (N=305)** |
| --- | --- | --- |
| Diabetic Clinic | 6 | 1.97% |
| Referral clinic | 8 | 2.62% |
| Medical Ward | 17 | 5.57% |
| Surgical Ward | 38 | 12.46% |
| Emergency | 185 | 60.66% |
| Unknown | 51 | 16.72% |
| **Total** | **305** | **100.00%** |

**Supplemental Table 3.** Initial cause of ulceration by category.

| **Cause of Ulceration** | **Count** | **Percentage (N=305)** |
| --- | --- | --- |
| Infected Plantar Warts | 1 | 0.33% |
| Infected Generalized Rash | 1 | 0.33% |
| Scabies | 2 | 0.66% |
| Shoes | 7 | 2.30% |
| Complication from Previous Procedure | 14 | 4.59% |
| Trauma | 135 | 44.26% |
| Unknown | 145 | 47.54% |
| **Total** | **305** | **100%** |

**Supplemental Table 4**. Pre-Operative laboratory results.

| **Pre-Operative Lab Test** | **Units** | **Count** | **Reference Range** | **Median** | **Mean** | **Min** | **Max** | **Standard Deviation** |
| --- | --- | --- | --- | --- | --- | --- | --- | --- |
| Sodium | mmol/L | 156 | 135-145 | 131 | 130.7 | 117 | 146 | 4.9 |
| Potassium | mmol/L | 157 | 3.5-5.3 | 4.2 | 4.2 | 2.7 | 7.1 | 0.6 |
| Haemoglobin | g/L | 293 | 130-180 (men)  120-160 (women) | 100 | 102.1 | 63 | 150 | 12.9 |
| Platelet Count | x10^9^/L | 284 | 150-400 | 388 | 406.0 | 34 | 1760 | 184.1 |
| Albumin | g/L | 48 | 35-50 | 25 | 33.4 | 8 | 419 | 57.5 |
| Creatinine | µmol/L | 110 | 60–110 (men)  45–90 (women) | 84.5 | 105.4 | 25 | 926 | 98.2 |
| Alkaline Phosphatase (ALP) | IU/L | 47 | 30-130 | 149 | 170.2 | 48 | 409 | 81.6 |
| Blood Urea Nitrogen | mmol/L | 98 | 2.5-7.8 | 5.76 | 8.8 | 1.6 | 54 | 8.3 |
| MCV | fl | 151 | 80-100 | 78.4 | 78.5 | 56 | 97 | 6.6 |
| MCH | pg | 152 | 27-31 | 27 | 30.6 | 20 | 342 | 32.1 |
| MCHC | g/L | 183 | 310-350 | 345 | 340.1 | 13.8 | 386 | 29.6 |
| WBC | x10^9^/L | 283 | 4-11 | 16 | 18.6 | 3.8 | 279.3 | 17.9 |
| WBC: Lymphocytes | % | 110 | 20-45 | 10.0 | 12.0 | 1.0 | 34.0 | 7.0 |
| WBC: Neutrophils | % | 109 | 40-75 | 83.0 | 81.0 | 56.0 | 96.0 | 9.0 |
| WBC: Eosinophils | % | 98 | 1-6 | 1.0 | 3.0 | 0.0 | 14.0 | 4.0 |
| WBC: Monocytes | % | 110 | 2-10 | 6.0 | 6.0 | 1.0 | 16.0 | 3.0 |
| WBC: Basophils | % | 93 | <1 | 0.0 | 3.0 | 0 | 14.0 | 4.0 |
